# Inequities in postnatal care in low- and middle-income countries: a systematic review and meta-analysis

**DOI:** 10.2471/BLT.14.140996

**Published:** 2015-04-01

**Authors:** Étienne V Langlois, Malgorzata Miszkurka, Maria Victoria Zunzunegui, Abdul Ghaffar, Daniela Ziegler, Igor Karp

**Affiliations:** a Alliance for Health Policy and Systems Research, World Health Organization, avenue Appia 20, 1211 Geneva, Switzerland.; b Research Centre of the University of Montreal Hospital Centre, Quebec, Canada.; c Library of the University of Montreal Hospital Centre, Quebec, Canada.; d Department of Epidemiology and Biostatistics, University of Western Ontario, London, Canada.

## Abstract

**Objective:**

To assess the socioeconomic, geographical and demographic inequities in the use of postnatal health-care services in low- and middle-income countries.

**Methods:**

We searched Medline, Embase and Cochrane Central databases and grey literature for experimental, quasi-experimental and observational studies that had been conducted in low- and middle-income countries. We summarized the relevant studies qualitatively and performed meta-analyses of the use of postnatal care services according to selected indicators of socioeconomic status and residence in an urban or rural setting.

**Findings:**

A total of 36 studies were included in the narrative synthesis and 10 of them were used for the meta-analyses. Compared with women in the lowest quintile of socioeconomic status, the pooled odds ratios for use of postnatal care by women in the second, third, fourth and fifth quintiles were: 1.14 (95% confidence interval, CI : 0.96–1.34), 1.32 (95% CI: 1.12–1.55), 1.60 (95% CI: 1.30–1.98) and 2.27 (95% CI: 1.75–2.93) respectively. Compared to women living in rural settings, the pooled odds ratio for the use of postnatal care by women living in urban settings was 1.36 (95% CI: 1.01–1.81). A qualitative assessment of the relevant published data also indicated that use of postnatal care services increased with increasing level of education.

**Conclusion:**

In low- and middle-income countries, use of postnatal care services remains highly inequitable and varies markedly with socioeconomic status and between urban and rural residents.

## ملخص

**الغرض:**

تقييم حالات الإجحاف الاجتماعية والاقتصادية والجغرافية والديمغرافية في الاستفادة من خدمات الرعاية الصحية بعد الولادة في البلدان المنخفضة الدخل والبلدان المتوسطة الدخل.

**الطريقة:**

أجرينا بحثاً في قواعد بيانات Medline وEmbase وكوكرين المركزية والمؤلفات غير الرسمية عن الدراسات التجريبية وشبه التجريبية والدراسات القائمة على الملاحظة التي تم إجراؤها في البلدان المنخفضة الدخل والبلدان المتوسطة الدخل. وقمنا بتلخيص الدراسات ذات الصلة من حيث النوع وأجرينا تحليلات وصفية للاستفادة من خدمات رعاية ما بعد الولادة وفقاً للمؤشرات المحددة للوضع الاجتماعي والاقتصادي والإقامة في بيئة حضرية أو ريفية.

**النتائج:**

تم إدراج ما مجموعه 36 دراسة في الملخص السردي وتم استخدام 10 منها لإجراء التحليلات الوصفية. ومقارنة بالنساء في الفئة الخميسية الأدنى للوضع الاجتماعي والاقتصادي، كانت نسب الاحتمال المجمعة لاستفادة النساء في الفئات الخميسية الثانية والثالثة والرابعة والخامسة من رعاية ما بعد الولادة على النحو التالي: 1.14 (فاصل الثقة 95 %، فاصل الثقة: من 0.96 إلى 1.34) و1.32 (فاصل الثقة: 95 %، فاصل الثقة: من 1.12 إلى 1.55) و1.60 (فاصل الثقة: 95 %، فاصل الثقة: من 1.30 إلى 1.98) و2.27 (فاصل الثقة: 95 %، فاصل الثقة: من 1.75 إلى 2.93)، على التوالي. ومقارنة بالنساء اللاتي يعشن في المناطق الريفية، كانت نسبة الاحتمال المجمعة لاستفادة النساء اللاتي يعشن في المناطق الحضرية من رعاية ما بعد الولادة 1.36 (فاصل الثقة: 95 %، فاصل الثقة: من 1.01 إلى 1.81). وأشار تقييم نوعي للبيانات المنشورة ذات الصلة كذلك إلى ازدياد الاستفادة من خدمات رعاية ما بعد الولادة بازدياد مستوى التثقيف.

**الاستنتاج:**

مازال هناك إجحاف كبير في البلدان المنخفضة الدخل والبلدان المتوسطة الدخل في الاستفادة من خدمات رعاية ما بعد الولادة، ويتباين ذلك بتباين الوضع الاجتماعي والاقتصادي وبين سكان المناطق الحضرية والريفية.

## 摘要

**目的:**

评估中低收入国家在使用产后卫生保健服务的社会经济学、地理学与人口学方面的不平等现象。

**方法:**

我们搜索了Medline、Embase、Cochrane Central数据库及灰色文献，查找在中低收入国家执行的试验、类试验和观察性研究。我们定性地总结了相关研究，并根据社会经济地位以及城市或农村居住等选定指标进行产后护理服务使用的综合分析。

**结果:**

总共36项研究被列入叙述合成，其中10项被用作荟萃分析。与社会经济地位五等分位数最低等的女性相比，排在第二、三、四、五等分位数的女性使用产后护理的合并优势比分别是：1.14（95%置信区间，CI：0.96-1.34）、1.32（95% CI：1.12-1.55）、1.60（95% CI：1.30-1.98）和2.27（95% CI：1.75-2.93）。与居住在农村环境的女性相比，居住在城市的女性使用产后护理的合并优势比是1.36（95% CI：1.01-1.81）。对相关公布数据的定性评估同样表明，产后护理服务使用的增加与教育程度成正比。

**结论:**

在中低收入国家，产后护理服务的使用依然处于极度不平等的阶段，因社会经济地位与城乡居民间的差异，产后护理的使用也明显不同。

## Résumé

**Objectif:**

Évaluer les inégalités socio-économiques, géographiques et démographiques en matière d'utilisation des soins de santé postnatals dans les pays à revenu faible et à revenu intermédiaire.

**Méthodes:**

Nous avons effectué une recherche bibliographique dans les bases de données Medline, Embase et Cochrane Central, ainsi que dans la littérature grise, pour trouver les études expérimentales, quasi-expérimentales et observationnelles qui ont été menées dans les pays à revenu faible et à revenu intermédiaire. Nous avons fait la synthèse qualitative des études pertinentes et effectué des méta-analyses de l'utilisation des services de soins postnatals en fonction des indicateurs choisis pour le statut socio-économique et la résidence dans les zones urbaines ou rurales.

**Résultats:**

Un total de 36 études ont été incluses dans la synthèse narrative et 10 d'entre elles ont été utilisées pour les méta-analyses. Par rapport aux femmes du quintile de statut socio-économique le plus bas, les rapports des cotes regroupés pour l'utilisation des soins postnatals par les femmes dans le deuxième, troisième quatrième et cinquième quintiles étaient de: 1,14 (intervalle de confiance à 95%, IC: 0,96-1,34) et 1,32 (IC à 95%: 1,12-1,55) et 1,60 (IC à 95%: 1,30-1,98) et 2,27 (IC à 95%: 1,75-2,93), respectivement. Par rapport aux femmes vivant en zones rurales, le rapport des cotes regroupé pour l'utilisation des soins postnatals par les femmes vivant en zones urbaines était de 1,36 (IC à 95%: 1,01-1,81). Une évaluation qualitative des données publiées pertinentes a également indiqué que l'utilisation des services de soins postnatals augmentait avec l’augmentation du niveau d'éducation.

**Conclusion:**

Dans les pays à revenu faible et à revenu intermédiaire, l'utilisation de services de soins postnatals reste très inéquitable et varie manifestement avec le statut socio-économique et entre les résidents urbains et ruraux.

## Резюме

**Цель:**

Оценить социально-экономическое, географическое и демографическое неравенство в получении услуг по уходу в постнатальном периоде в странах с низким и средним уровнем доходов.

**Методы:**

Мы изучили базы данных Medline, Embase и Cochrane Central, а также не индексированную в медицинских базах данных литературу, посвященную экспериментальным, квазиэкспериментальным и обсервационным исследованиям, которые проводились в странах с низким и средним уровнем доходов. Мы обобщили качественные результаты соответствующих исследований и выполнили метаанализ использования услуг по уходу в постнатальном периоде в соответствии с отобранными показателями социально-экономического статуса и проживания в городской или сельской местности.

**Результаты:**

В нарративный синтез было включено в общей сложности 36 исследований; 10 из них использовались для метаанализа. Сравнив показатели женщин, входящих в нижний квинтиль социально-экономического статуса, мы получили следующие обобщенные отношения шансов на получение услуг по уходу в постнатальном периоде для женщин, входящих во второй, третий, четвертый и пятый квинтили: 1,14 (доверительный интервал (ДИ) 95%: 0,96–1,34), 1,32 (ДИ 95%: 1,12–1,55), 1,60 (ДИ 95%: 1,30–1,98) и 2,27 (ДИ 95%: 1,75–2,93) соответственно. По сравнению с женщинами, проживающими в сельской местности, обобщенные отношения шансов на получение услуг по уходу в постнатальном периоде для женщин, проживающих в городской местности, составили 1,36 (ДИ 95%: 1,01–1,81). Результаты качественной оценки соответствующих опубликованных данных также свидетельствуют о том, частота использования услуг по уходу в постнатальном периоде уменьшается с понижением уровня образования.

**Вывод:**

В странах с низким и средним уровнем доходов сохраняется существенное неравенство в получении услуг по уходу в постнатальном периоде, которое зависит главным образом от социально-экономического статуса и отличается среди городских и сельских жителей.

## Resumen

**Objetivo:**

Evaluar las desigualdades socioeconómicas, geográficas y demográficas en el uso de los servicios de salud de atención posnatal en países de ingresos bajos y medianos.

**Métodos:**

Se buscaron estudios experimentales, cuasiexperimentales y observacionales que se habían llevado a cabo en países de ingresos bajos y medianos en las bases de datos Medline, Embase y Cochrane Central, así como en la literatura gris. Se resumieron los estudios relevantes cualitativamente y se realizaron metaanálisis sobre el uso de los servicios de atención posnatal según indicadores seleccionados del nivel socioeconómico y residencia en un entorno urbano o rural.

**Resultados:**

Se incluyó un total de 36 estudios en la síntesis narrativa, 10 de los cuales se utilizaron para los metaanálisis. En comparación con las mujeres del quintil más bajo del nivel socioeconómico, las razones de posibilidades agrupadas del uso de atención posnatal de las mujeres en el segundo, tercer, cuarto y quinto quintiles fueron: 1,14 (intervalo de confianza del 95 %, IC: 0,96–1,34), 1,32 (IC del 95 %: 1,12–1,55), 1,60 (IC del 95 %: 1,30–1,98) y 2,27 (IC del 95 %: 1,75–2,93), respectivamente. En comparación con las mujeres que viven en entornos rurales, la razón de posibilidades agrupada del uso de atención posnatal por mujeres que viven en entornos urbanos fue 1,36 (IC del 95 %: 1,01–1,81). Una evaluación cualitativa de los datos relevantes publicados indicó además que el uso de servicios de atención posnatal ha aumentado mediante el aumento del nivel de educación.

**Conclusión:**

En los países de ingresos bajos y medianos, el uso de servicios de atención posnatal sigue siendo muy desigual y varía notablemente con el nivel socioeconómico y entre los residentes urbanos y rurales.

## Introduction

Each year an estimated 289 000 women die worldwide from complications related to pregnancy, childbirth or the postnatal period^1^ and up to two thirds of such maternal deaths occur after delivery.^2^^,^^3^ Poor outcomes of maternal and neonatal care also include 2.9 million neonatal deaths per year.^4^ Of the maternal and neonatal deaths that occur globally, 99% occur in low- and middle-income countries.^1^^,^^5^

According to the World Health Organization (WHO), the postnatal period begins immediately after childbirth and lasts six weeks.^6^ In low-income countries, almost 40% of women experience complications after delivery and an estimated 15% develop potentially life-threatening problems.^7^ Postnatal care services are a fundamental element of the continuum of essential obstetric care – which also includes antenatal care and skilled birth attendance – that decreases maternal and neonatal morbidity and mortality in low- and middle-income countries.^8^^,^^9^ Compared with other maternal and infant health services,^10^ coverage for postnatal care tends to be relatively poor. Increasing such coverage has been highlighted as a priority.^11^ In the Democratic Republic of the Congo, for example, at least 93% of pregnant women receive antenatal care and skilled birth attendance but only 35% of birthing women receive postnatal care.^12^ In Kenya, fewer than 20% of women use postnatal care services.^13^ In 2014, WHO recommended that a mother and her newborn child should receive postnatal care within 24 hours of the birth and then at least three more times – i.e. at least on day three after the birth, in the second week after the birth and six weeks after the birth.^14^ Postnatal care services can be defined as preventive care practices and assessments that are designed to identify and manage or refer complications for both the mother and the neonate. Typically, such services include an integrated package of routine maternal and neonatal care as well as extra care for neonates that are considered particularly vulnerable because, for example, they are preterm, have a low birth weight, are small for gestational age or have mothers infected with human immunodeficiency virus (HIV).^15^ Possible postnatal interventions for the mother include: (i) iron and folic acid supplementation for at least three months; (ii) screening for – and treatment of – infection, haemorrhage, thromboembolism, postnatal depression and other conditions; (iii) prophylactic antibiotics given to women who have a third- or fourth-degree perineal tear; and (iv) counselling on early and exclusive breastfeeding, nutrition, birth spacing and family planning options – including any available contraception.^14^^,^^16^^,^^17^ Possible interventions for the neonate include: (i) care of the umbilical cord (ii) special care for preterm, low-birth-weight and HIV-infected neonates;^14^^,^^15^^,^^18^ (iii) screening and treatment of infections and postnatal growth restriction; (iv) assessment of factors predisposing to infant anaemia;^19^ and (v) teaching the mother to seek additional care for her neonate if she notices danger signs such as convulsions or problems with feeding.^14^

Low use of postnatal care services is associated with lack of education, poverty and limited access to health-care facilities.^2^ However, these associations have not been assessed systematically. We therefore conducted a systematic review of the relevant evidence from low- and middle-income countries, to inform policy-making, help strengthen health systems and increase access to – and use of – postnatal care services.

## Methods

We followed guidelines for systematic reviews from the Cochrane Collaboration^20^ and a standardized methodology described in an explicit protocol.^21^ The review was registered with the Prospero database (registration number: CRD42013004661) and results were reported according to the Preferred Reporting Items for Systematic Reviews and Meta-Analyses (PRISMA) statement.^22^

### Literature search

To identify the studies of interest, we searched the Medline, Embase and Cochrane Central databases and grey literature for relevant medical subject headings and keywords. We focused on articles published between 1 January 1960 and 31 May 2013 in English, French, Spanish, Portuguese and Chinese and were assisted by an expert librarian. Our search strategy combined terms related to postnatal or postpartum care, use or accessibility, determinants or inequities and low- or middle-income countries. Our full search strategy is detailed in Appendix A (available at: https://dl.dropboxusercontent.com/u/28446882/Appendix%20A.pdf). To identify further data that might be useful, we also checked the reference lists of the articles found to be of potential interest, visited institutional web sites and contacted the authors of some of the articles of interest and other experts in the field.

### Inclusion criteria

We retrieved data from experimental, quasi-experimental and observational studies of women aged 15–49 years, that had been implemented in low- or middle-income countries as defined by the World Bank.^23^ The primary outcome of interest was the use of postnatal care services – i.e. at least one follow-up visit in the 42 days post-childbirth. We included studies in which the potential socioeconomic, geographical and/or demographic determinants of the use of postnatal care had been assessed. The potential socioeconomic determinants that we investigated were socioeconomic status, occupation and education. We investigated distance and travel time to a health centre and place of residence – i.e. urban or rural – as potential geographical determinants and ethnicity, marital status, religion and immigration status as potential demographic determinants. We analysed data from studies that included at least one association measure – such as a frequency ratio or difference – or the result of at least one statistical test in which use of postnatal care had been compared across two or more categories. We included relative comparisons to a reference group (e.g. concentration indexes) and absolute comparisons (e.g. slope indexes of inequality). In some relevant studies, a concentration index was used to measure the relationship between accumulated proportions of mothers ranked by their socioeconomic status against the cumulative proportion of postnatal care use. In these studies, a positive value for the index indicates that rich households have greater coverage than poor households, a negative index indicates that poor households have greater coverage than rich households and zero values for the index that coverage is independent of socioeconomic status. Other studies used a slope index of inequality to estimate the absolute difference in percentage postnatal care coverage between individuals at the top and bottom of the socioeconomic status scale. In such studies, a high slope index of inequality would have indicated great inequity in coverage.

### Data extraction

The eligibility of each study identified in the initial screening was assessed by two reviewers using a standardized form with explicit inclusion and exclusion criteria. There was a high level of agreement between the reviewers (Cohen’s kappa,^24^ *κ*: 0.92). Data were extracted with a standardized data collection form^21^ that had been pilot tested on a random sample of studies. We collected data on country, setting, year of publication, study design, sample size, population attributes, outcome definition, comparison groups, point estimates and precision measures.

### Quality assessment

Two individuals, working independently, assessed the scientific quality of each selected study using the Effective Public Health Practice Project’s quality assessment tool for quantitative studies – after extending the criteria for selection bias assessment.^25^ Scientific quality was categorized as high, moderate or low if, respectively, the risk of bias in the study results was considered to be very low, low or high. The level of agreement between the two assessors of quality was good (*κ*: 0.75). Discrepancies in the assessment of eligibility or scientific quality were resolved in discussions with an experienced researcher.

### Data synthesis

Evidence tables were generated to summarize the selected studies and results descriptively. We conducted a qualitative synthesis of the findings. We also conducted a meta-analysis of selected studies that provided a comparable classification of the outcome and determinants of interest. For this purpose, we also required either estimates of the standard errors for the association measure or confidence intervals that allowed us to derive such estimates.^21^ Many of the studies included in the systematic review had to be excluded from the meta-analysis because of differences in the classification or definition of determinants. We pooled the association measures for socioeconomic status and geography, as represented by socioeconomic status quintile and an indicator of urban/rural place of residence, respectively. We assessed heterogeneity of these results using Cochran’s *Q* test^26^and the *I*^2^ statistic. We used random-effects meta-analysis models when heterogeneity was statistically significant (*P* > 0.1) and *I*^2^ was moderate or high according to the criteria of Higgins et al.^27^ We conducted sensitivity analyses by removing studies deemed to be of low quality or potential outliers.^28^^,^^29^ We assessed publication bias in the meta-analyses with funnel plots. Data analysis was performed using Stata version 12.0 (StataCorp LP, College Station, United States of America).

## Results

**Fig. 1:**
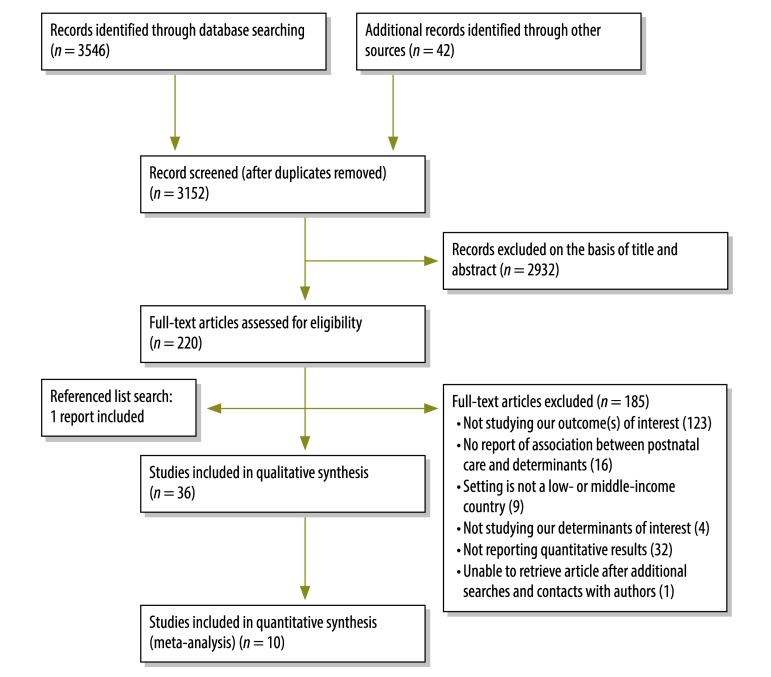
Flowchart for the selection of studies on potential determinants of the use of postnatal care in low- and middle-income countries

Our initial search produced 3152 articles of potential interest and articles describing 36 studies^2^^,^^7^^,^^11^^,^^12^^,^^17^^,^^30^^–^^60^ contributed to our qualitative synthesis of evidence (Fig. 1). Data from 10 of the studies were included in the meta-analysis.^7^^,^^30^^–^^32^^,^^34^^,^^35^^,^^39^^,^^41^^,^^51^^,^^60^ The 36 studies included in our qualitative synthesis of evidence comprised two randomized controlled trials, three quasi-experimental studies, two cohort and 28 cross-sectional studies, and one investigation of 31 demographic and health surveys. Of these 36 studies, 11 were conducted in low-income countries, 24 in middle-income countries and one in both low- and middle-income countries. Three, 26 and six of the 36 studies were deemed to be of high, moderate and low scientific quality, respectively. A lack of information on methodology prevented the assessment of the scientific quality of one study included in the qualitative synthesis (Table 1, available at: http://www.who.int/bulletin/volumes/93/4/14-140996).

**Table 1:** Characteristics of studies included in the systematic review on the use of postnatal care services in low- and middle-income countries

Study	Country, year	Design	*n*	Quality^a^	Setting
Abbas and Walker (1986)^57^	Jordan, 1979	Cross-sectional national population census, with multi-stage random cluster sampling	1 765	Low	At the time of the study, 72% of women in rural areas lived more than 5 km from a maternal and child health clinic. The corresponding values for women in the three main cities and other urban areas were 7% and 10%, respectively. Most women (53%) reported that they had not received any education on pregnancy or child health
Abel Ntambue et al. (2012)^12^	Democratic Republic of the Congo, 2010	Cross-sectional study	1 762	Low	Study based in the city of Lubumbashi – the administrative centre of Katanga province and the second most populated city in the country. At the time of the study, the city had an estimated population of 1 415 835 and was divided into health-care zones that were mainly urban and where almost all health services were operational and easily accessible
Agha (2011)^30^	Pakistan, 2008–2009	Quasi-experimental before-and-after study, with no control group. Intervention: voucher scheme for obstetric services	1 423	Moderate	Study area was DG Khan city – a small city located in southern Punjab, in one of the poorest districts of Pakistan
Agha and Carton (2011)^31^	Pakistan, 2011	Cross-sectional representative household survey	2 018	Moderate	At the time of the study, financial barriers to use of maternal health services remained substantial in rural areas of Jhang district, Pakistan. About 38% of women who did not have their last birth in a health facility cited the high cost of care as the reason for not doing so
Amin et al. (2010)^32^	Bangladesh, 2003–2006	Cross-sectional household survey	1 212	Moderate	Study in 128 rural villages in three of the six divisions of Bangladesh: Chittagong, Dhaka and Rajshahi. Study villages were outside the catchment areas of nongovernmental health centres and could be considered remote. None of the villages was served by a health service
Anson (2004)^33^	China, 1996–1999	Cross-sectional representative household survey	4 273	Moderate	Survey of 288 villages in the rural northern province of HeBei. At the time of the study, privatization of rural health services and the costs of unauthorized births presented considerable barriers to the use of maternal care services The share of public funding for maternal health services had declined considerably and this had led to increases in out-of-pocket expenditure
Anwar et al. (2008)^34^	Bangladesh, 2006	Cross-sectional community survey	2 164	Moderate	Survey in rural and periurban areas. The mean distance between home and the nearest government hospital was 6.2 km. Government services were provided free of charge
Babalola and Fatusi (2009)^35^	Nigeria, 2000–2005	Cross-sectional nationally representative household survey in 36 states	2 148	Moderate	Nigeria's maternal mortality ratio is higher than the regional average and there is wide regional disparity in health status among Nigeria's diverse and multi-ethnic settings
Baqui et al. (2008)^36^	India, 2001 –2005	Quasi-experimental clustered before-and-after study with control group. Intervention: community nutrition and health government programme facilitated by CARE-India	14 952	Moderate	Study in two districts of rural Uttar Pradesh –India’s largest state and one of the most disadvantaged. Barriers remain with regards to accessibility and cost of services
Chakraborty et al. (2002)^37^	Bangladesh, 1992–1993	Cohort study with multi-stage random sampling	1 020	Moderate	In Bangladesh, four out of five women experience at least one morbidity during their index pregnancy and puerperium
Chatterjee and Paily (2011)^56^	India, 2005–2006	Cross-sectional nationally representative family health survey	131 596	Low	At the time of the study, only 20.3% of expenditure on health came from the government and 77.4% came from the patients’ pockets. There was very little insurance coverage available for maternity services in India, particularly in rural areas and user fees remain the norm for postnatal services
Dhaher et al. (2008)^2^	West Bank and Gaza Strip, 2006	Cross-sectional study	264	Moderate	Study based in three clinics located in the three largest cities in the West Bank: northern Jenin, central Ramallah and southern Hebron. Clinics provide most of the reproductive health services and are referral clinics for surrounding villages and camps
Dhakal et al. (2007)^38^	Nepal, 2006	Cross-sectional study	150	Moderate	The study was conducted in two Village Development Committee (VDC) areas of Kathmandu district. These were slightly more developed than a typical VDC in Nepal
Halder et al. (2007)^39^	Bangladesh, 2004	Cross-sectional study, with multi-stage cluster sampling	4 838	Moderate	Although reproductive health services had been expanded in the two decades prior to the study, such services were available largely to the women in urban centres. Use of such services remained very low among the poor and in underserved rural areas
Iyoke et al. (2011)^40^	Nigeria, 2007–2008	Cross-sectional study	371	Low	Study based in two main tertiary hospitals in the south-eastern city of Enugu: University of Nigeria Teaching Hospital and Enugu State Teaching Hospital. At the time of the study, the estimated population of Enugu was 635 451 and most residents were civil servants or traders
Jat et al. (2011)^41^	India, 2007–2008	Cross-sectional study – a nationwide household survey following a multi-stage stratified systematic sampling design	15 782	Moderate	At the time of the study, only 26.7% of Indians in Madhya Pradesh state resided in urban areas and the state was one of the poorest six states of India. About 38% of the state’s population was living below the poverty line in 2004–2005. In 2008, there were 270 community health centres, 1149 primary health centres and 8834 health sub-centres in the state. These provided preventive and curative health-care services in rural areas. The state also had a huge network of private health-care facilities, although these were mainly concentrated in urban areas
Kabakian-Khasholian and Campbell (2005)^17^	Lebanon, 2000–2001	Randomized controlled trial. Intervention versus placebo	378	High	At the time of the study, Lebanon lacked an organized health-care system and the public health-care sector only played a minor role. Four private hospitals – two in Beirut and two in the Bekaa region – were selected. All were privately owned and one in Beirut was a teaching hospital. These hospitals are located in urban areas but attract women from the suburbs of Beirut and from surrounding villages in the Bekaa. Similar to other facilities in Lebanon, postpartum practices in these hospitals are characterized by a short postpartum hospital stay and an absence of home follow-up
Liu et al. (2011)^42^	China, 2005	Cross-sectional study with multi-stage random sampling	14 112	Moderate	Study based in western China, where most areas are mountainous with poor economic conditions and health services, and scarce information is available on the use of maternal health-care services
Mahabub-Ul-Anwar et al. (2006)^43^	Bangladesh, 2004	Cross-sectional survey	848	Low	Study based in rural areas where the government provides reproductive health services through its Health and Family Welfare Centres. At the time of the study, more than 60% of the population of these areas did not have access to basic health care and more than 80% of women received no postnatal care. In theory, the rural population had free access to primary health care, family planning and reproductive health services. However, the non-availability of service providers at government facilities in rural areas was a major problem. In the private health sector, the poor were not protected by any subsidized pricing structure
Matijasevich et al. (2009)^11^	Brazil, 2004	Cohort study	3 497	High	The study was based in the southern city of Pelotas, when the city had a population of about 340 000 – 93% of them living in the urban area. Brazil’s publicly funded health-care system offers free access to postnatal care for every woman
Mistry et al. (2009)^44^	India, 1998–1999	Cross-sectional study, with multi-stage sampling design	11 648	Moderate	Study based in rural villages, most of which had low economic status and poor public health infrastructures
Mullany et al. (2007)^45^	Nepal, 2003–2004	Randomized controlled trial	442	High	Study based in an urban area
Mullany et al. (2008)^59^	Myanmar, 2006–2007	Cross-sectional population-based sample with two-stage cluster sampling	2 252	Low	At the time of the study, about 560 000 individuals had been internally displaced within Shan, Karenni, Karen and Mon states, along Myanmar’s eastern border. Myanmar has one of the world’s least functioning health systems and within the conflict zones, there is practically no functioning public health sector and the performance indicators for obstetric care are even lower than national mean values
Okafor (1991)^46^	Nigeria, 1988–1989	Cross-sectional study	498	Moderate	Study based in 25 communities in the Udi local government area, when the area had a general hospital, a cottage hospital and six maternity centres. The surveyed women resided in rural towns
Rahman et al. (2011)^7^	Bangladesh, 2007	Cross-sectional study, with stratified, multi-stage cluster sampling	10 996	Moderate	At the time of the study, two thirds of the young mothers in Bangladesh lived in rural areas, more than one in six were uneducated and over three quarters were in unpaid jobs
Rai et al. (2012)^60^	Nigeria, 2003–2008	Cross-sectional study, with equal-probability systematic sampling	2 434	Moderate	At the time of the study, 23% of female Nigerians aged 15–19 years had begun childbearing. Hardly any married women in Nigeria used modern methods of contraception. Over two thirds had their first pregnancy when aged less than 18 years and 46% of women aged 20–49 years had been married by the time they reached 18 years
Ram and Singh (2006)^47^	India, 1998–2002	Cross-sectional household survey	11 454	Moderate	Study set in Uttar Pradesh, where, at the time of the study, around 90% of deliveries were conducted at home and nearly half the deliveries were only assisted by family or friends
Sarma and Rempel (2007)^48^	India, 1995–1996	Cross-sectional, nationally representative household survey	16 592	Moderate	At the time of the study, distance to the nearest source of postnatal care consistently had a negative effect on a woman’s registration for – and use of – such care. Access to a bus service was an important positive determinant for the use of maternal health-care services in rural areas
Sharma et al. (2007)^49^	Nepal, 1996–2001	Quasi-experimental before-and-after study with no control group but representative samples of the Nepalese population	7 788	Moderate	The Safe Motherhood Programme was implemented in Nepal in 1997. It was launched as a priority programme during the 1997–2002 plan period, with the aim of increasing women’s access to health care and raising their status
Singh et al. (2012)^50^	India, 2007–2008	Cross-sectional nationwide household survey following a multi-stage stratified systematic sampling design	93 416	Moderate	At the time of the study, infant mortality rates in most Indian states appeared to be stabilizing rather than falling. Overall, there were 212 maternal deaths per 100 000 live births but levels of maternal mortality varied widely across the states. Although pregnant women were offered cash incentives to give birth in a health facility, there was no similar scheme in place to promote postnatal care
Singh et al. (2012)^51^	India, 2005–2006	Cross-sectional study with representative samples from all 29 states	3 599	Moderate	In 2005, the Indian government launched the National Rural Health Mission to improve health-system performance and people’s health status in rural areas. A conditional cash-transfer scheme was also launched to promote institutional deliveries among women in rural areas
Stupp et al. (1994)^52^	Belize, 1991	Cross-sectional area-probability household survey with two stages of selection	979	Moderate	In rural areas of Belize, the tropical terrain and lack of roads – especially all-weather roads – reduce access to health care for rural women. Some ethnic groups may be particularly disadvantaged because they mainly live in rural settings
Tang and Li (2008)^53^	China, 1998–2003	Cross-sectional study with stratified cluster sampling	462	Low	The study was based in poor regions of Sichuan province
Titaley et al (2009)^58^	Indonesia, 2002–2003	Cross-sectional survey with systematic stratified random sampling	15 553	Low	Study conducted in 26 of Indonesia’s 30 provinces
Zere et al. (2010)^54^	Namibia, 2006–2007	Cross-sectional study	9 804	Low	Namibia has one of the highest levels of income inequality in the world. At the time of the study, almost all Namibian women paid for delivery – mainly in cash but also in kind. However, 85% each paid less than the equivalent of 7.0 United States dollars

^a^ Studies were considered to be of high, moderate and low quality if there was judged to be a very low, low and high risk of bias in the results, respectively.

### Socioeconomic determinants

#### Socioeconomic status

**Fig. 2:**
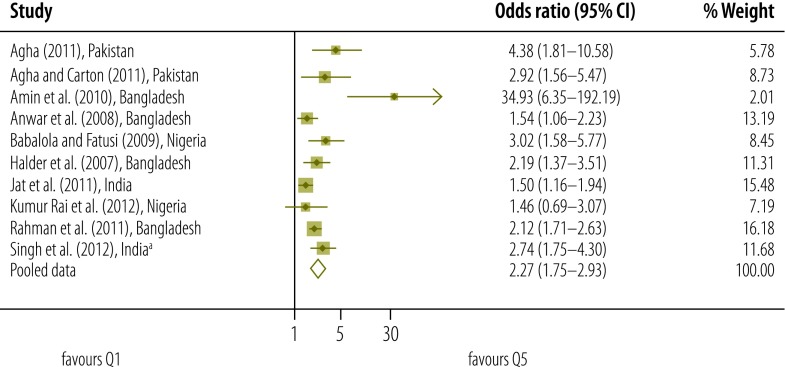
Odds ratio for the association between socioeconomic status and use of postnatal care services; quintile 5 versus quintile 1 (reference)

^a^ Singh et al.^51^

Notes: In each study, women were assigned to one of five socioeconomic status quintiles, from the highest (Q5) to the lowest (Q1). Each odds ratio is an estimate for a comparison between the women in Q5 and those in Q1, with the latter used as the reference category.

**Fig. 3:**
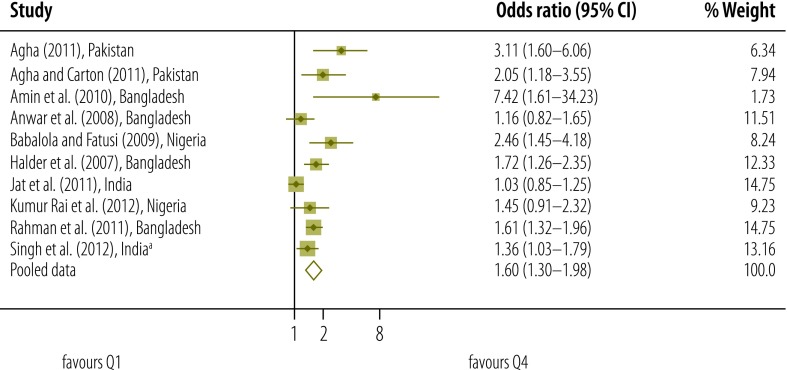
Odds ratio for the association between socioeconomic status and use of postnatal care services; quintile 4 versus quintile 1 (reference)

^a^ Singh et al.^51^

Notes: In each study, women were assigned to one of five socioeconomic status quintiles, from the highest (Q5) to the lowest (Q1). Each odds ratio is an estimate for a comparison between the women in Q4 and those in Q1, with the latter used as the reference category.

**Fig. 4:**
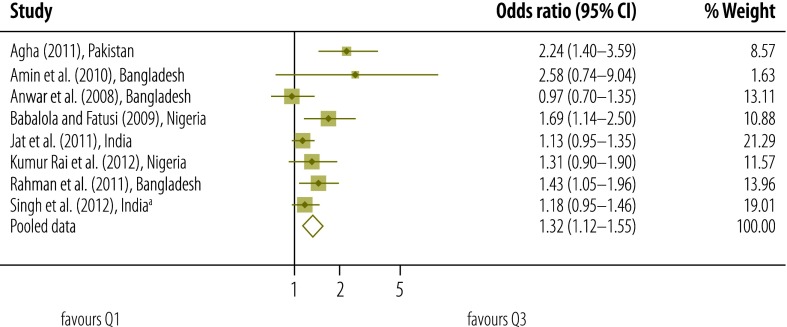
Odds ratio for the association between socioeconomic status and use of postnatal care services; quintile 3 versus quintile 1 (reference)

^a^ Singh et al.^51^

Notes: In each study, women were assigned to one of five socioeconomic status quintiles, from the highest (Q5) to the lowest (Q1). Each odds ratio is an estimate for a comparison between the women in Q3 and those in Q1, with the latter used as the reference category.

**Fig. 5:**
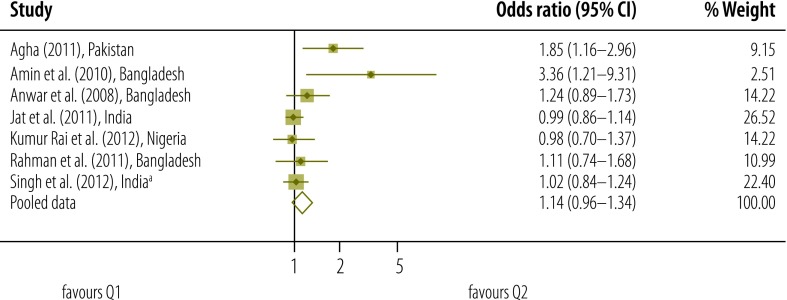
Odds ratio for the association between socioeconomic status and use of postnatal care services; quintile 2 versus quintile 1 (reference)

^a^ Singh et al.^51^

Notes: In each study, women were assigned to one of five socioeconomic status quintiles, from the highest (Q5) to the lowest (Q1). Each odds ratio is an estimate for a comparison between the women in Q2 and those in Q1, with the latter used as the reference category.

Our qualitative assessment of relevant studies indicates that there was a gradient in the use of postnatal care according to socioeconomic status – as measured on different scales (Table 2, available at: http://www.who.int/bulletin/volumes/93/4/14-140996).^2^^,^^7^^,^^11^^,^^12^^,^^17^^,^^30^^–^^54^^,^^60^ Results of our meta-analysis that included data on socioeconomic status from studies of moderate quality also indicated that the higher the socioeconomic status of the mother, the more likely she was to access postnatal care (Fig. 2, Fig. 3, Fig. 4 and Fig. 5).

**Table 2:** Socioeconomical determinants for the use of postnatal care services in low- and middle-income countries

Study	Adjusted	Comparison groups	Odds ratio^a^
Abel Ntambue et al. (2012)^12^	No	Woman’s occupation, with housewife used as reference	Estimated, for use of PNC for no more than 7 days after the birth, for salesperson (0.8; 95% CI: 0.1–1.1), agricultural worker (0.6; 95% CI: 0.1–1.0) and public service worker (0.8; 95% CI: 0.4–1.3). The corresponding estimates for use of PNC for no more than 28 days after the birth were 0.9 (0.6–1.2), 0.7 (0.2–1.1) and 0.9 (0.7–1.4), respectively. The corresponding estimates for use of PNC for no more than 42 days were 1.0 (0.8–1.3), 0.8 (0.5–1.2) and 1.1 (0.7–1.6), respectively
		Woman’s level of education, with secondary used as reference	Estimated – for the non-use of PNC for the first 7 days after the birth – for primary (1.1; 95% CI: 0.8–1.6) and university (1.0; 95% CI: 0.7–1.5) levels. The corresponding estimates for the non-use of PNC for the first 28 days after the birth were 1.1 (0.8–1.5) and 1.0 (0.8–1.4), respectively. The corresponding estimates for the non-use of PNC for the first 42 days after the birth were 1.2 (0.9–1.5) and 1.4 (1.0–1.9), respectively
Agha (2011)^30^	Yes	Wealth quintiles, with the fifth/poorest quintile used as reference	Estimated for fourth (1.31; NS), third (2.24; *P* < 0.001), second (3.11; *P* < 0.001) and first (4.38; *P* < 0.001) quintiles
		Exposure to voucher scheme versus no exposure	4.98 (*P* < 0.001)
		Woman’s level of education, with none used as reference	Estimated for primary (1.73; *P* < 0.01), middle (1.33; NS) secondary (2.59; *P* < 0.001) and higher (3.97; *P* < 0.001) levels of education
Agha and Carton (2011)^31^	Yes	Wealth quintiles, with the first/poorest quintile used as reference	Estimated for second (1.85; *P* < 0.01), third (1.39; *P* < 0.01), fourth (2.05; *P* < 0.01) and fifth (2.92; *P* < 0.001) quintiles
		Woman’s level of education, with none used as reference	Estimated for less than primary (1.23; NS), completed primary (1.39; NS), middle (1.90; *P* < 0.01) and higher (1.84; *P* < 0.01) levels of education
Amin et al. (2010)^32^	Yes	Wealth quintiles, with the first/poorest quintile used as reference	Estimated for second (3.36; 95% CI:1.20–9.39), third (2.58; 95% CI: 0.73–9.06), fourth (7.42; 95% CI: 1.61–34.29) and fifth (34.93; 95% CI: 6.30–193.64) quintiles
		Credit group member versus non-member	1.53 (95% CI: 0.64–3.67)
		Woman’s level of education, with none used as reference	Estimated for 1–5 years (0.60; 95% CI: 0.25–1.42) or longer (2.14; 95% CI: 0.93–4.93) in education
		Partner’s level of education, with none used as reference	Estimated for 1–5 years (0.94; 95% CI: 0.42–2.08) or longer (0.34; 95% CI: 0.11–1.04) in education
		Partner’s occupation, with agriculture used as reference	Estimated for unskilled labour (1.18; 95% CI: 0.40–3.52) and skilled occupations (0.97; 95% CI: 0.36–2.65)
Anson (2004)^33^	Yes	Household per-capita income	1.01 (NS)
		Woman’s years of schooling	1.10 (*P* < 0.001)
		Woman’s occupation, categorized as white-collar or other, with “other” used as reference	2.17 (*P* < 0.001)
Anwar et al. (2008)^34^	Yes	Asset quintile, with first/lowest used as reference	Estimated for second (1.24; 95% CI: 0.89–1.72), third (0.97; 95% CI: 0.69–1.37), fourth (1.16; 95% CI: 0.81–1.65) and fifth (1.54; 95% CI: 1.05–2.25) quintiles
		Woman’s level of education, with none used as reference	Estimated for 1–4 (1.25; 95% CI: 0.89–1.76), 5–9 (0.90; 95% CI: 0.65–1.24) and more (1.19; 95% CI: 0.75–1.87) years of education
		Husband’s level of education, with none used as reference	Estimated for 1–4 (1.38; 95% CI: 0.99–1.92), 5–9 (1.06; 95% CI: 0.79–1.42) and more (1.32; 95% CI: 0.90–1.92) years of education
Babalola and Fatusi (2009)^35^	Yes	Household socioeconomic status, with very poor used as reference	Estimated for poor (1.01; NS), moderately rich (1.69; *P* < 0.01), rich (2.46; *P* < 0.001) and very rich (3.02; *P* < 0.001) households
		Woman’s level of education, with none used as reference	Estimated for primary (1.65; *P* < 0.001), secondary (2.06; *P* < 0.001) and higher (3.50; *P* < 0.001) levels of education
Baqui et al. (2008)^36^	No	Wealth quintiles, with effect on home visits for PNC investigated	Change in absolute concentration index calculated for intervention (−0.2253; 95% CI: −0.2894 to −0.1612) and comparison (0.0104; 95% CI: −0.0761 to 0.0969) districts
Chakraborty et al. (2002)^37^	Yes	Economic status, with good versus poor used as reference	Postnatal care by doctor/nurse/family-welfare visitor OR = 0.883 (0.276–2.823) Postnatal care by other OR = 1.009 (0.599–1.700)
		Mother’s education, with some versus none used as reference	Postnatal care by doctor/nurse/family-welfare visitor OR = 0.949 (0.387–2.328) Postnatal care by other OR = 1.143 (0.760–1.719)
		Husband’s occupation, with business/service versus other used as reference	Postnatal care by doctor/nurse/family-welfare visitor OR = 1.937 (0.809–4.634) Postnatal care by other: OR = 2.096 (1.409–3.118)
		Women’s gainful employment, with yes versus no used as reference	Postnatal care by doctor/nurse//family-welfare visitor OR = 0.873 (0.341–2.236) Postnatal care by other: OR = 0.686 (0.473–0.996)
Dhaher et al. (2008)^2^	Yes	Level of education of woman and husband, with education of both above secondary level used as reference	Estimated for couples in which only the man (0.9; 95% CI: 0.3–2.2) or woman (95% CI: 1.5; 0.6–3.4) or neither individual (1.9; 95% CI: 0.8–4.5) was educated above secondary level
Dhakal et al. (2007)^38^	Yes	Woman’s occupation, with farmer used as reference	Estimated for housewife (6.28; 95% CI: 2.00–19.69) and other (3.06; 95% CI: 0.27–34.64) occupations
		Husband’s occupation, with farmer used as reference	Estimated for males who have worked abroad and/or in the formal sector (0.83; 95% CI: 0.27–2.53) and for other non-farmers (0.15; 95% CI: 0.03–0.85)
		Woman’s level of education, with illiterate used as reference	Estimated for primary (1.25; 95% CI: 0.45–3.42) and secondary (6.49; 95% CI: 2.5–17.2) levels
		Husband’s level of education, with illiterate used as reference	Estimated for primary (1.32; 95% CI: 0.28–6.92) and secondary (6.33; 95% CI: 1.55–29.95) levels
Halder et al. (2007)^39^	Yes	Wealth index quintiles, with the first/poorest used as reference	Estimated for second (1.223; NS), third (1.107; NS), fourth (1.723; *P* < 0.001) and fifth (2.188; *P* < 0.001) quintiles
		Woman’s level of education, with none used as reference	Estimated for primary (1.445; *P* < 0.001) and higher (1.935; *P* < 0.001) levels
		Partner’s occupation, with farmer used as reference	Estimated for agricultural and non-agricultural labourers (1.299; *P* < 0.05), semi-skilled labourers (1.204; NS), those who run small businesses (1.149; NS) and those in more well paid occupations (1.447; *P* < 0.05)
Halder et al. (2007)^39^	Yes	Wealth index quintiles, with the first/poorest used as reference	Estimated for second (1.223; NS), third (1.107; NS), fourth (1.723; *P* < 0.001) and fifth (2.188; *P* < 0.001) quintiles
Iyoke et al. (2011)^40^	No	Income group, categorized as income earner or other, with other used as reference	8.40 (*P* = 0.37)
		Woman’s level of education, with primary or less used as reference	Estimated for secondary (0.75), tertiary (1.38) and post-tertiary (1.23) education, with an overall *P*-value of 0.15
Jat et al. (2011)^41^	Yes	Socioeconomic status, with the poorest used as reference	Estimated for the poor (0.99; 95% CI: 0.85–1.14), moderately rich (1.13; 95% CI: 0.95–1.35), rich (1.03; 95% CI: 0.84–1.26) and richest (1.50; 95% CI: 1.16–1.93)
		Woman’s poverty index, categorized as holders or non-holders of a below-the-poverty-line ration card, with the holders used as reference	0.88 (95% CI: 0.79–0.98)
		Woman’s level of education, with illiterate used as reference	Estimated for primary (1.00; 95% CI: 0.86–1.15), middle (1.17; 95% CI: 0.99–1.37) and higher (1.39; 95% CI: 1.14–1.70) levels
		Woman’s occupation, with unemployed used as reference	Estimated for agricultural workers (0.92; 95% CI: 0.81–1.04) and professional, service or production workers (0.97; 95% CI: 0.77–1.23)
		Husband’s level of education, with illiterate used as reference	Estimated for primary (1.03; 95% CI: 0.87–1.21), middle (0.98; 95% CI: 0.83–1.15) and higher (1.14; 95% CI: 0.96–1.35) levels
Kabakian-Khasholian and Campbell (2005)^17^	Yes	Intervention: information booklet in Arabic, covering the correct breastfeeding position; maternal health problems; importance of the postnatal check-up at 6 weeks after the birth, father’s role in the postnatal period and family planning	Relative risk estimated to be 2.8 (95% CI: 2.2– 3.4)
		Woman’s level of education, with below secondary used as reference	Relative risks estimated for secondary (1.8; 95% CI: 1.1– 2.5) and university (2.7; 95% CI: 2.1– 3.4) levels
Liu et al. (2011)^42^	Yes	Wealth index, categorized as: poor, middle or rich, with poor used as reference	Estimated for the middle (1.28; 95% CI: 1.01–1.63) and rich (1.36; 95% CI: 1.03–1.80) categories
		Woman’s level of education, with primary used as reference	Estimated for secondary (1.00; 95% CI: 0.85–1.18) and high school (1.13; 95% CI: 0.85–1.49) levels
		Husband’s level of education, with primary used as reference	Estimated for secondary (0.89; 95% CI: 0.76–1.06) and high school (0.75; 95% CI: 0.48–1.16) levels
Mahabub-Ul-Anwar et al. (2006)^43^	No	Wealth group quintile, with first/poorest used as reference	Estimated for the second (1.01), third (1.34), fourth (1.47) and fifth (2.31) quintiles
Matijasevich et al. (2009)^11^	Yes	Family income quintiles, with the fifth/richest used as reference	Estimated for the first (2.61; 95% CI: 1.85–3.66), second (2.17; 95% CI: 1.55–3.05), third (2.02; 95% CI: 1.44–2.82) and fourth (1.51; 95% CI: 1.07–2.13) quintiles, with an overall *P*-value of less than 0.001
		Insurance scheme, categorized as public or private, with private used as reference	3.08 (1.99–4.79)
		Woman’s years of schooling, with over 9 years used as reference	Estimated for 0–4 (2.64; 95% CI: 2.01–3.48) and 5–8 (2.04; 95% CI: 1.64−2.54) years, with an overall *P*-value of less than 0.001
Mistry et al. (2009)^44^	Yes	Woman’s standard of living, categorized as low, medium or high, with low used as reference	Estimated for the medium (1.21; 95% CI: 1.06–1.39) and high (1.84; 95% CI: 1.49–2.28) categories
		Woman’s employment status, categorized as currently employed or unemployed, with unemployed used as reference	0.93 (95% CI: 0.82–1.06)
		Woman’s years of education	1.07 (95% CI: 1.06–1.09)
		Partner’s years of education	1.00 (95% CI: 0.98–1.01)
Mullany et al. (2007)^45^	Yes	Intervention: antenatal health education sessions on birth preparedness and use of maternal health care, with non-intervention group used as reference	Relative risks estimated for a couples group (1.29; 95% CI: 1.04–1.60) and a women-only group (1.03; 95% CI: 0.82–1.31)
Okafor (1991)^46^	Yes	Woman’s years of education	1.10 (*P* < 0.01)
Rahman et al. (2011)^7^	Yes	Woman’s wealth index, with poorest used as reference	Estimated – in a comparison of skilled PNC versus unskilled or no such care – for the poor (1.11; 95% CI: 0.67–1.51), middle (1.43; 95% CI: 1.11–2.06), richer (1.61; 95% CI: 1.34–1.97) and richest (2.12; 95% CI: 1.68–2.58). In a comparison of PNC on 1 or 2 days with more days of PNC, the corresponding values were 1.24 (0.83–1.86), 1.75 (0.94–1.82), 1.84 (1.23–2.76) and 2.08 (1.68–2.58), respectively
		Woman’s level of education, with none used as reference	Estimated – in a comparison of skilled PNC versus unskilled or no such care – for incomplete (1.33; 95% CI: 0.78–1.49) and complete primary (1.41; 95% CI: 0.81–1.68), incomplete secondary (1.53; 95% CI: 1.12–2.00) and higher (2.03; 95% CI: 1.42–2.86) levels. In a comparison of PNC on 1 or 2 days with more days of PNC, the corresponding values were 1.07 (0.82–1.62), 1.17 (0.94–1.45), 1.51 (1.11–2.06) and 1.84 (1.23–2.76), respectively
		Woman’s occupation, categorized as paid job or unpaid job, with unpaid used as reference	Estimated – in a comparison of skilled PNC versus unskilled or no such care – as 1.22 (95% CI: 0.91–1.44). In a comparison of PNC on 1 or 2 days with more days of PNC, the corresponding value was 1.14 (0.83–1.56)
		Husband’s occupation, with manual labour used as reference	Estimated – in a comparison of skilled PNC versus unskilled or no such care – for agricultural workers and the self-employed (1.02; 95% CI: 0.84–1.77), professional, technical and managerial workers (2.22; 95% CI: 1.62–2.81) and other occupations (1.93; 95% CI: 1.23–2.67). In a comparison of PNC on 1 or 2 days with more days of PNC, the corresponding values were 1.11 (0.85–1.56), 1.61 (1.32–1.97) and 1.14 (0.83–1.56), respectively
Rai et al. (2012)^60^	Yes	Wealth quintile, with first/poorest used as reference	Estimated for second (0.976; 95% CI: 0.705–1.352), third (1.310; 95% CI: 0.908–1.889), fourth (1.453; 95% CI: 0.907–2.326) and fifth (1.465; 95% CI: 0.688–3.121) quintiles
		Woman’s work status, with not working used as reference	Estimated for working at home (1.112; 95% CI: 0.828–1.492) and away from home (1.132; 95% CI: 0.809–1.584)
		Woman’s level of education, with none used as reference	Estimated for primary but below middle (1.534; 95% CI: 1.067–2.206) and for secondary and above (1.116; 95% CI: 0.706–1.765)
		Husband’s level of education, with none used as reference	Estimated for primary but below middle (1.405; 95% CI: 0.990–1.993) and for secondary and above (1.638; 95% CI: 1.137–2.361)
Ram and Singh (2006)^47^	Yes	Standard of living index, categorized as low, medium or high, with low used as reference	Estimated for medium (1.232; *P* < 0.05) and high (1.096; NS)
		Respondent’s level of education, categorized as literate or illiterate, with illiterate used as reference	0.971 (NS)
Sarma and Rempel (2007)^48^	Yes	Woman’s level of education, with illiterate used as reference	Estimated for rural women who had achieved primary (1.277; *P* < 0.01), secondary (1.453; *P* < 0.01) or higher (2.081; *P* < 0.01) levels. The corresponding values for urban women were 1.321 (*P* < 0.01), 1.715 (*P* < 0.01) and 2.413 (*P* < 0.01), respectively
Sharma et al. (2007)^49^	Yes	Household economic status, categorized as possessing household durable goods or services or otherwise, with otherwise used as reference	1.30 (*P* < 0.001)
		Woman’s employment, with not employed used as reference	Estimated for manual workers (0.63; NS), agricultural workers and the self-employed (0.53; *P* < 0.001) and service and other workers (0.65; *P* < 0.001)
		Woman’s level of education, with none used as reference	Estimated for primary (0.96; NS) and higher (1.83; *P* < 0.001) levels
Singh et al. (2012)^50^	Yes	Concentration index	Estimated for home (0.027; *P* < 0.001) and facility (0.027; *P* < 0.001) births among mothers who received any check-ups within 48 hour of the birth. Corresponding estimates were made for neonates who were checked within 24 hours of birth – 0.182 (*P* < 0.001) and 0.054 (*P* < 0.001), respectively – or checked at least twice within first 10 days of life – 0.073 *(P <* 0.001) and 0.061 (*P* < 0.001), respectively – as well as for neonates who were checked at government facilities – 0.015 (*P* < 0.001) and 0.166 (*P* < 0.001), respectively – or private facilities – 0.157 (*P* < 0.001) and 0.255 (*P* < 0.001), respectively.
Singh et al. (2012)^51^	Yes	Wealth quintile, with poorest used as reference	Estimated for poorer (1.021; 95% CI: 0.841–1.239), middle (1.183; 95% CI: 0.956–1.464), richer (1.360; 95% CI: 1.038–1.783) and richest (2.741; 95% CI: 1.729–4.347)
		Woman’s level of education, with illiterate used as reference	Estimated for literate but below primary (1.417; 95% CI: 1.112–1.806), primary (1.588; 95% CI: 1.309–1.927) middle (1.912; 95% CI: 1.501–2.434) and higher (1.917; 95% CI: 1.399–2.627) levels
		Husband’s level of education, with illiterate used as reference	Estimated for literate but below primary (1.059; 95% CI: 0.804–1.394), primary (1.286; 95% CI: 1.042–1.587), middle (1.070; 95% CI: 0.855–1.339) and higher (1.026; 95% CI: 0.811–1.297) levels
Stupp et al. (1994)^52^	Yes	Woman’s years of education, with 1–7 used as reference	Estimated for 8 (1.37; *P* < 0.05) and 9–12 (2.16; *P* < 0.01) years
		Woman’s working status, categorized as currently working or not working, with not working used as reference	1.30 (*P* < 0.10)
Tang and Li (2008)^53^	No	Annual family per capita income, with a value of less than 1000 yuan used as reference	Estimated for 1000–2999 (1.59), 3000–5000 (1.49) and more than 5000 (1.59) yuan
		Woman’s level of education, with “illiterate or semi-literate” used as reference	Estimated for primary school (1.19), junior high school (1.40), high school (1.75) and higher (1.34) levels
Zere et al. (2010)^54^	No	Household wealth index	Concentration index estimated to be 0.0835 (95% CI: 0.0823–0.0847)

CI: confidence interval; NS: not significant; PNC: postnatal care.

^a^ Unless another association measure is indicated. Odds ratios were estimated for the use of postnatal care services unless indicated otherwise.

Meta-analysis was used to derive pooled adjusted odds ratios (OR) from 10 studies and a total of 136 431 women. For each quintile of socioeconomic status, the *Q* test gave a significant result and the *I*^2^ statistic fell between 50% and 75% – indicating moderate heterogeneity.^27^ When the lowest quintile (Q1) was used as the reference, the pooled OR for the highest quintile (Q5) was 2.27 (95% confidence interval, CI: 1.75–2.93). The corresponding OR for Q4, Q3 and Q2 were lower, at 1.60 (95% CI: 1.30–1.98; *I*^2^: 70%), 1.32 (95% CI: 1.12–1.55; *I*^2^: 50%) and 1.14 (95% CI: 0.96–1.34; *I*^2^: 52%), respectively.

In a sensitivity analysis, we removed the potentially atypical data reported by Amin et al.^32^ The pooled OR for Q5, Q4, Q3 and Q2 – with Q1 used as the reference – were reduced to 2.09 (95% CI: 1.70–2.56), 1.55 (95% CI: 1.27–1.90), 1.30 (95% CI: 1.10–1.54) and 1.08 (95% CI: 0.95–1.24), respectively.

The data in a report^55^ included in the systematic review showed concentration indexes and slope indexes of inequality for use of postnatal care in 31 countries (Table 3). For the low-income countries, the mean concentration index was 0.23 and the mean slope index of inequality was 53%. The corresponding values for the middle-income countries were 0.18 and 61%, respectively. In Pakistan, exposure to a voucher scheme led to significant increase in the use of postnatal care (OR: 4.98; *P* < 0.001).^30^

**Table 3:** Socioeconomic inequities in postnatal care coverage

Country, source of data	Value for postnatal care within 2 days of birth	
	Concentration index	Slope index of inequality (percentage points)
**Low-income countries**		
Bangladesh (DHS 2007)	0.371	50.0
Benin (DHS 2006)	0.100	49.5
Cambodia (DHS 2010)	0.152	54.9
Democratic Republic of the Congo (DHS 2007)	0.114	49.2
Haiti (DHS 2005)	0.382	66.3
Kenya (DHS 2008)	0.244	67.0
Liberia (DHS 2007)	0.195	54.1
Madagascar (DHS 2008)	0.202	53.3
Malawi (DHS 2010)	0.053	25.7
Mali (DHS 2006)	0.206	58.0
Nepal (DHS 2006)	0.414	54.7
Niger (DHS 2006)	0.526	59.5
Sierra Leone (DHS 2008)	0.106	27.2
Uganda (DHS 2006)	0.195	51.5
United Republic of Tanzania (DHS 2010)	0.189	60.6
Zimbabwe (DHS 2005)	0.146	60.1
**Middle-income countries**		
Azerbaijan (DHS 2006)	0.080	42.3
Bolivia (DHS 2008)	0.143	65.5
Congo (DHS 2005)	0.086	46.3
Egypt (DHS 2008)	0.133	56.7
Ghana (DHS 2008)	0.196	70.9
India (DHS 2005)	0.338	77.3
Indonesia (DHS 2007)	0.208	66.5
Lesotho (DHS 2009)	0.168	61.7
Nigeria (DHS 2008)	0.392	83.6
Pakistan (DHS 2006)	0.281	64.9
Peru (DHS 2004)	0.131	67.8
Philippines (DHS 2008)	0.189	64.0
Sao Tome and Principe (DHS 2008)	0.048	25.3
Swaziland (DHS 2006)	0.105	49.8
Zambia (DHS 2007)	0.241	70.8

DHS: Demographic and Health Survey.

Data source: adapted from *Countdown to 2015. Maternal, newborn & child survival. Building a future for women and children. The 2012 report*.^55^

#### Level of education

Our qualitative assessment of studies indicated marked variations in the use of postnatal care according to the level of education of the women investigated – or their partners (Table 2). Compared to women who had received no formal education, women who had attended primary education were more likely to use postnatal care^30^^,^^35^^,^^48^^,^^50^^,^^60^ and women who had completed secondary school were the most likely to access postnatal care.^7^^,^^17^^,^^38^^,^^39^^,^^41^^,^^48^^,^^49^^,^^51^ In three studies, the duration of maternal schooling was found to be positively correlated with postnatal care use.^33^^,^^44^^,^^46^ Compared with other women, those with husbands who had completed secondary school also appeared more likely to use postnatal care.^38^^,^^41^^,^^60^ In Lebanon, an educational intervention to emphasize the importance of postnatal care led to a marked increase in the use of such care (relative risk: 2.8; 95% CI: 2.2–3.4).^17^ Inconsistent classification of education status prevented us from performing a meta-analysis of these apparent determinants of the use of postnatal care.

#### Occupation

The income-earning occupations of women and their husbands appear to influence the women’s use of postnatal care (Table 2). For example, women married to men with professional, technical or managerial occupations were more likely to use postnatal care than women married to manual labourers (OR: 2.22; 95% CI: 1.62–2.81).^7^ Similarly, women married to men with well paid jobs were more likely to use postnatal care than women married to farmers (OR: 1.45; *P* < 0.05).^39^ In China, women with so-called white-collar occupations were more likely to use postnatal care than other women (OR: 2.17; *P* < 0.001).^33^ Inconsistent classification of occupation impeded any corresponding meta-analysis.

### Geographical determinants

**Fig. 6:**
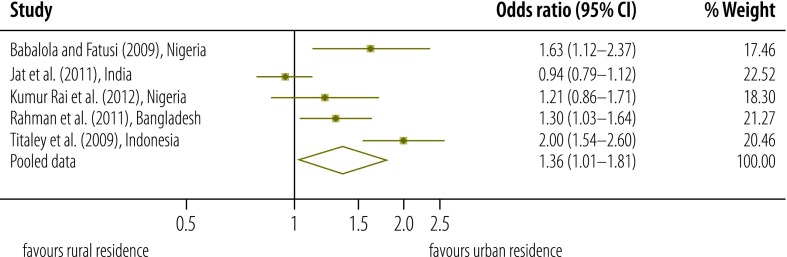
Odds ratio for the association between place of residence and use of postnatal care services

Notes: In each study, women were categorized as urban or rural residents. Each odds ratio is an estimate for a comparison between the urban and rural women, with the latter used as the reference category.

A qualitative assessment of the evidence indicated that postnatal care was more commonly used by women living in urban areas than by their rural counterparts (Table 4).^7^^,^^35^^,^^39^^,^^41^^,^^49^^,^^52^^,^^56^^–^^58^^,^^60^ Our meta-analysis of this trend was based on five studies and a total of 46 913 women.^7^^,^^35^^,^^41^^,^^58^^,^^60^ As a *Q* test gave a significant result (*P* < 0.001) and *I*^2^ was 83.7%, heterogeneity was considered high.^27^ With women in rural areas used as the reference, our initial estimate of the pooled OR for use of postnatal care by women residing in urban areas was 1.36 (95% CI: 1.01–1.81; Fig. 6). After removing the study deemed to be of low quality,^58^ the estimated pooled OR became 1.21 (95% CI: 0.95–1.53). In several studies included in our systematic review, distance to the nearest health facility was also found to be associated with use of postnatal care services. In India, for example, the relevant OR for distances of 2–5 and at least 6 km – with a distance of less than 2 km used as the reference – were 0.80 (95% CI: 0.67–0.95) and 0.64 (95% CI: 0.50–0.83), respectively.^44^ In rural areas of India, the presence of a bus service has been found to increase the use of postnatal care services (OR: 1.18; *P* < 0.01).^48^

**Table 4:** Geographical determinants for the use of postnatal care services in low- and middle-income countries

Study	Adjusted	Comparison groups	Odds ratio^a^
Abbas and Walker (1986)^57^	No	Place of residence categorized as urban or rural, with rural used as reference	Estimated – for the non-use of PNC – as 1.40
Agha (2011)^30^	Yes	Travel time to nearest health facility, categorized as no more than 5 minutes or more than 5 minutes, with the longer time used as reference	1.81 (*P* < 0.001)
Agha and Carton (2011)^31^	Yes	Travel time to nearest health facility, categorized as no more than 15 minutes or more than 15 minutes, with the longer time used as reference	1.13 (NS)
Anson (2004)^33^	Yes	Distance to county hospital	0.99 (*P* < 0.01)
Anwar et al. (2008)^34^	Yes	Distance to hospital, categorized as more than 5 km or 0–5 km, with 0–5 km used as reference	1.21 (95% CI: 0.98–1.50)
Babalola and Fatusi (2009)^35^	Yes	Place of residence categorized as urban or rural, with rural used as reference	1.63 (*P* < 0.01)
Chakraborty et al. (2002)^37^	Yes	Distance to health facility, categorized as at least 1 km or less than 1 km, with less than 1 km used as reference	Estimated for care provided by doctor, nurse or family welfare visitor (0.659; 95% CI: 0.277–1.567) and care provided by other individual (1.111; 95% CI: 0.744–1.658)
Chatterjee and Paily (2011)^56^	No	Place of residence categorized as urban or rural, with rural used as reference	3.83
Halder et al. (2007)^39^	Yes	Place of residence categorized as urban or rural, with rural used as reference	1.176 (NS)
Jat et al. (2011)^41^	Yes	Place of residence categorized as urban or rural, with rural used as reference	0.94 (95% CI: 0.78–1.11)
Liu et al. (2011)^42^	Yes	Altitude of residence above sea level, with no more than 500 m used as reference	Estimated for 501–1500 (0.49; 95% CI: 0.25–0.97) and more than 1500 m (0.54; 95% CI: 0.30–0.98)
Mistry et al. (2009)^44^	Yes	Distance to health facility, with less than 2 km used as reference	Estimated for 2–5 (0.80; 95% CI: 0.67–0.95) and at least 6 km (0.64; 95% CI: 0.50–0.83)
Mullany et al. (2008)^59^	No	Forced displacement or relocation in prior 12 months or otherwise, with otherwise used as reference	0.40 (95% CI: 0.13–1.28)
Okafor (1991)^46^	Yes	Distance from service	0.99 (*P* < 0.01)
Rahman et al. (2011)^7^	Yes	Place of residence, categorized as urban or rural, with urban used as reference	Estimated as 0.77 (95% CI: 0.53–0.84) in a comparison of skilled PNC versus unskilled or no such care and as 0.52 (95% CI: 0.42–0.65) in a comparison of PNC on 1 or 2 days with more days of PNC
		Distance to health facility, with less than 1 km used as reference	Estimated as 1.23 (95% CI: 0.91–1.72). in a comparison of skilled PNC versus unskilled or no such care and as 1.10 (95% CI: 0.84–1.43) in a comparison of PNC on 1 or 2 days with more days of PNC
Rai et al. (2012)^60^	Yes	Place of residence categorized as urban or rural, with rural used as reference	1.212 (95% CI: 0.861–1.706)
Ram and Singh (2006)^47^	Yes	Distance to transport facility, categorized as 0–2 km or more than 2 km, with the longer distance used as reference	0.947 (NS)
Sarma and Rempel (2007)^48^	Yes	Distance to health facility, with less than 2 km used as reference	Estimated for 2–5 (0.777; *P* < 0.01), 5–10 (0.746; *P* < 0.01) and more than 10 km (0.751; *P* < 0.01)
		Availability of bus service in rural areas, with none available used as reference	1.178 (*P* < 0.01)
Sharma et al. (2007)^49^	Yes	Place of residence categorized as urban or rural, with rural used as reference	1.24
Singh et al. (2012)^51^	Yes	Region of residence, with south used as reference	Estimated for north (0.219; 95% CI: 0.165–0.291), central (0.089; 95% CI: 0.070–0.113), east (0.157; 95% CI: 0.127–0.193), north-east (0.068; 95% CI: 0.043–0.107) and west regions (0.309; 95% CI: 0.238–0.400).
Stupp et al. (1994)^52^	Yes	Place of residence categorized as rural or not rural, with not rural used as reference	0.83 (NS)
Titaley et al. (2009)^58^	ND	Place of residence, categorized as urban or rural, with urban used as reference	Estimated – for non-use of PNC – as 2.00 (95% CI: 1.54–2.60)

CI: confidence interval; ND: not determined; NS: not significant; PNC: postnatal care.

^a^ Unless another association measure is indicated. Odds ratios were estimated for the use of postnatal care services unless indicated otherwise.

### Demographic determinants

#### Religion

In one study, use of postnatal care services was higher among Muslim women than among Christian women (OR: 2.01; 95% CI: 1.24–3.25).^60^ In contrast, in another study, Muslim women seemed less likely to use such services than their non-Muslim counterparts (OR: 0.77; 95% CI: 0.61–1.34).^7^ In Nepal, compared with Hindu women, Buddhist women were less likely to use postnatal care services (OR: 0.25; *P* < 0.001).^49^ Overall, our systematic review of relevant studies revealed no clear trend in the use of such services according to religion (Table 5).

**Table 5:** Demographical determinants for the use of postnatal care services in low- and middle-income countries

Study	Adjusted	Comparison groups	Odds ratio^a^
Abel Ntambue et al. (2012)^12^	No	Woman’s marital status, with married used as reference	Estimated for the non-use of PNC within 7 (2.8; 95% CI: 0.9–14.1), 28 (1.7; 95% CI: 0.8–3.5) or 42 days of the birth (1.3; 95% CI: 0.8–2.3)
Anwar et al. (2008)^34^	Yes	Woman’s religion, categorized as Muslim or other, with Muslim used as reference	0.87 (95% CI: 0.57–1.33)
Babalola and Fatusi (2009)^35^	Yes	Woman’s ethnic group, with Hausa used as reference	Estimated for Yoruba (1.57; NS), Igbo (2.10; *P* < 0.05), Fulani (1.22; NS), Kanuri (0.97; NS) and other groups (1.55; *P* < 0.10)
Dhakal et al. (2007)^38^	No	Woman’s ethnic group, with Brahmin-Chhetri used as reference	Estimated for Tamang (0.15; 95% CI: 0.05–0.44) and other groups (1.03; 95% CI: 0.31–3.38).
Iyoke et al. (2011)^40^	No	Woman’s marital status, with single used as reference	1.40 (*P* = 0.50)
Jat et al. (2011)^41^	Yes	Proportion of population in woman’s district of residence considered tribal, with a value of more than 50% used as reference	Estimated for 26–50% (0.60; 95% CI: 0.26–1.35) and 0–25% (0.52; 95% CI: 0.23–1.16)
		Woman’s caste, with scheduled tribe used as reference	Estimated for scheduled (0.85; 95% CI: 0.70–1.03) and other castes (0.92; 95% CI: 0.77–1.08)
		Woman’s religion, with Hindu used as reference	Estimated for Muslim (0.81; 95% CI: 0.63–1.03) and other (1.46; 95% CI: 0.75–2.83)
Liu et al. (2011)^42^	Yes	Woman’s ethnic group, categorized as Han or minority, with minority used as reference	0.92 (95% CI: 0.74–1.15)
Matijasevich et al. (2009)^11^	Yes	Woman’s skin colour, categorized as black/mixed or white, with white used as reference	1.37 (95% CI: 1.16–1.63)
Mistry et al. (2009)^44^	Yes	Woman’s social group, with “other” used as reference	Estimated for scheduled caste (0.98; 95% CI: 0.83–1.16), scheduled tribe (0.64; 95% CI: 0.52–0.79) and other so-called backward classes (0.95: 95% CI: 0.82–1.09)
		Woman’s religion, with Hindu used as reference	Estimated for Muslim (1.10; 95% CI: 0.90–1.35) and other (1.11; 95% CI: 0.90–1.37)
Mullany et al. (2008)^59^	No	Woman’s ethnic group, with Karen or Karenni used as reference	Estimated for Shan or Mon (8.38; 95% CI: 4.12–17.03)
Rahman et al. (2011)^7^	Yes	Woman’s religion, categorized as Muslim or non-Muslim, with non-Muslim used as reference	Estimated as 0.77 (95% CI: 0.61–1.34) in a comparison of skilled PNC versus unskilled or no such care and as 0.72 (95% CI: 0.66–1.03) in a comparison of PNC on 1 or 2 days with more days of PNC
Rai et al. (2012)^60^	Yes	Woman’s religion, categorized as Muslim or Christian, with Christian used as reference	2.008 (95% CI: 1.239–3.252)
		Woman’s ethnic group, with Igbo or Yoruba used as reference	Estimated for Hausa, Fulani or Kanuri (0.585; 95% CI: 0.250–1.371) and other groups (95% CI: 0.904; 0.408–2.003)
Ram and Singh (2006)^47^	Yes	Woman’s social group, with scheduled caste or scheduled tribe used as reference	Estimated for other so-called backward classes (1.039; NS) and other ethnicities (1.081; NS)
		Woman’s religion, categorized as Muslim or Hindu, with Hindu used as reference	1.164 (NS)
Sarma and Rempel (2007)^48^	Yes	Woman’s caste, categorized as either upper caste or scheduled caste or tribe, with upper caste used as reference	Estimated separately for rural (1.026; NS) and urban areas (0.960; NS)
Sharma et al. (2007)^49^	Yes	Woman’s religion, with Hindu used as reference	Estimated for Buddhist (0.25; *P* < 0.001), Muslim (1.25; NS) and other (0.41; *P* < 0.05)
Singh et al. (2012)^51^	Yes	Woman’s religion, with Hindu used as reference	Estimated for Muslim (0.877; 95% CI: 0.686–1.121) and other (0.918; 95% CI: 0.618–1.365)
		Woman’s social group, with other used as reference	Estimated for scheduled castes (0.693; 95% CI: 0.555–0.865), scheduled tribes (0.706; 95% CI: 0.545–0.915) and other so-called other backward classes (0.584; 95% CI: 0.481–0.709)
Stupp et al. (1994)^52^	Yes	Woman’s origins, categorized as immigrant or native, with native used as reference	1.31 (NS)
		Woman’s religion, categorized as Catholic or non-Catholic, with non-Catholic used as reference	0.97 (NS)
		Woman’s ethnicity and language, with Creole used as reference	Estimated for Spanish-speaking (0.64, *P* < 0.01) and non-Spanish-speaking Mestizo (1.37; NS), Garifuna (1.25; NS) and Mayan-speaking (0.71; NS) and non-Mayan-speaking Maya (0.42; *P* < 0.01)

CI: confidence interval; NS: not significant; PNC: postnatal care.

^a^ Unless another association measure is indicated. Odds ratios were estimated for the use of postnatal care services unless indicated otherwise.

#### Ethnicity

In India, women belonging to the lower social groups – i.e. those belonging to scheduled castes (OR: 0.69; 95% CI: 0.55–0.86), scheduled tribes (OR: 0.71; 95% CI: 0.54–0.91) or other so-called backward classes (OR: 0.58; 95% CI: 0.48–0.71) – were found to be less likely to use postnatal care services than those belonging to upper castes (Table 5).^51^ Although we found statistically significant differences in the use of postnatal care services according to the ethnicity of the women investigated, our systematic review revealed no clear trend in the use of such services according to whether the woman involved belonged to a minority or majority group.^11^^,^^35^^,^^38^^,^^44^^,^^51^^,^^52^^,^^59^

## Discussion

We have systematically reviewed studies assessing inequities in the use of postnatal care services in low- and middle-income countries. We found strong and consistent evidence indicating that the use of such services was relatively high among women with high socioeconomic status and among more educated women. In general, women with high socioeconomic status belong to those households that can afford the medical, non-medical and opportunity costs of postnatal care.^8^ In addition, such women may be relatively empowered and have more autonomy than their poorer counterparts.^61^Educated women are considered to have relatively good access to – and management of – health service information, and relatively accurate and detailed perceptions of diseases and their complications and treatments.^8^^,^^62^ There also seems to be an independent association between a woman’s use of maternal services and her partner’s education.^63^

In addition to increasing household income, employment can increase awareness and modify a person’s behaviour, through social and community interactions.^49^ However, in low- and middle-income countries, there seems to be no clear and consistent association between a woman’s income-generating employment and her use of postnatal care services. A woman in gainful employment may still have no control over any of her household’s finances. In addition, a woman’s economic activity may also be poverty-induced, only seasonal and/or relatively poorly remunerated.^8^^,^^64^

Compared with women living in rural areas, urban women have generally better access to postnatal care services as well as other advantages of urban life, such as greater exposure to health-promotion programmes.^60^^,^^65^ In many rural areas, improvements in the numbers of primary health care facilities, the provision of postnatal care services of high quality and public transportation are required. Although the relationship between ethnicity and use of postnatal care services appears complex, there are some ethnicities, such as India’s lower castes, that often seem to be disadvantaged.^66^

We found insufficient homogeneous classification of data to conduct meta-analyses for occupation or level of education. Our meta-analysis for place of residence may have been weakened by the suboptimal precision of a between-studies variance estimate.^67^ Despite these limitations, our study indicates that the use of postnatal care remains highly inequitable according to socioeconomic status, education and geographical access to health facilities. There are several research and knowledge gaps that need to be filled. For example, we need research to further understand health-seeking behaviours and to inform policy-makers. As most maternal deaths occur during the postnatal period, primary research on postnatal care services should be prioritized. Further research on the contextual and systems-level determinants of the use of such services and the effectiveness of strategies to improve the coverage and quality of postnatal care is also needed. It remains unclear if the number and timing of postnatal consultations recommended by WHO are optimal and achievable in every setting.^14^ It also remains to be determined if postnatal care at home can be made as effective and cost-effective as similar care provided by health facilities.^14^ We need both community-level interventions to promote the use of postnatal care services and health systems interventions to improve the supply of affordable and quality services – including, but not limited to, alleviation of user-fees and the promotion of postnatal care by health professionals. Strengthening the effectiveness and responsiveness of systems for health-care delivery^68^ will also catalyse access to – and use of – postnatal and other obstetric care services. In the current and future elaboration of universal health coverage and equity schemes in low- and middle-income countries, due consideration should be provided to postnatal care services.
